# Effect of starvation on brain glucose metabolism and ^18^F-2-fluoro-2-deoxyglucose uptake: an experimental in-vivo and ex-vivo study

**DOI:** 10.1186/s13550-018-0398-0

**Published:** 2018-06-11

**Authors:** Ambra Buschiazzo, Vanessa Cossu, Matteo Bauckneht, Annamaria Orengo, Patrizia Piccioli, Laura Emionite, Giovanna Bianchi, Federica Grillo, Anna Rocchi, Francesco Di Giulio, Francesco Fiz, Lizzia Raffaghello, Flavio Nobili, Silvia Bruno, Giacomo Caviglia, Silvia Ravera, Fabio Benfenati, Michele Piana, Silvia Morbelli, Gianmario Sambuceti, Cecilia Marini

**Affiliations:** 1 Department of Health Science, Nuclear Medicine Unit, University of Genoa, Genoa, Italy; 2 Nuclear Medicine Unit, Polyclinic San Martino Hospital, Largo R. Benzi 10, 16132 Genoa, Italy; 3 Cell Biology Unit, Polyclinic San Martino Hospital, Genoa, Italy; 4 Animal Facility, Polyclinic San Martino Hospital, Genoa, Italy; 5 Oncology Lab, IRCCS Giannina Gaslini, Genoa, Italy; 6 Pathology, Department of Integrated Surgical and Diagnosic Sciences (DISC), University of Genoa, Genoa, Italy; 7 Center for Synaptic Neuroscience and Technology, Istituto Italiano di Tecnologia (IIT), Genoa, Italy; 8 Department of Experimental Medicine, University of Genoa, Genoa, Italy; 9 Nuclear Medicine Unit, Department of Radiology, Uni-Klinikum, Tuebingen, Germany; 10 Clinical Neurology, Polyclinic San Martino Hospital, Genoa, Italy; 11 Department of Neuroscience (DINOGMI), University of Genoa, Genoa, Italy; 12 Department of Mathematics (DIMA), University of Genoa, Genoa, Italy; 13 Department of Pharmacy, Biochemistry Laboratory, University of Genoa, Genoa, Italy; 14 SPIN Institute, CNR, Genoa, Italy; 15 CNR Institute of Molecular Bioimaging and Physiology (IBFM), Milan, Italy

**Keywords:** Brain metabolism, PET/CT imaging, FDG, Starvation, Neuroimaging

## Abstract

**Background:**

The close connection between neuronal activity and glucose consumption accounts for the clinical value of 18F-fluoro-2-deoxyglucose (FDG) imaging in neurodegenerative disorders. Nevertheless, brain metabolic response to starvation (STS) might hamper the diagnostic accuracy of FDG PET/CT when the cognitive impairment results in a severe food deprivation.

**Methods:**

Thirty six-week-old BALB/c female mice were divided into two groups: “control” group (*n* = 15) were kept under standard conditions and exposed to fasting for 6 h before the study; the remaining “STS” mice were submitted to 48 h STS (absence of food and free access to water) before imaging. In each group, nine mice were submitted to dynamic micro-PET imaging to estimate brain and skeletal muscle glucose consumption (C- and SM-MRGlu*) by Patlak approach, while six mice were sacrificed for ex vivo determination of the lumped constant, defined as the ratio between CMRGlu* and glucose consumption measured by glucose removal from the incubation medium (*n* = 3) or biochemical analyses (*n* = 3), respectively.

**Results:**

CMRGlu* was lower in starved than in control mice (46.1 ± 23.3 vs 119.5 ± 40.2 nmol × min^−1^ × g^−1^, respectively, *p* < 0.001). Ex vivo evaluation documented a remarkable stability of lumped constant as documented by the stability of GLUT expression, G6Pase activity, and kinetic features of hexokinase-catalyzed phosphorylation. However, brain SUV in STS mice was even (though not significantly) higher with respect to control mice. Conversely, a marked decrease in both SM-MRGlu* and SM-SUV was documented in STS mice with respect to controls.

**Conclusions:**

STS markedly decreases brain glucose consumption without altering measured FDG SUV in mouse experimental models. This apparent paradox does not reflect any change in lumped constant. Rather, it might be explained by the metabolic response of the whole body: the decrease in FDG sequestration by the skeletal muscle is as profound as to prolong tracer persistence in the bloodstream and thus its availability for brain uptake.

## Background

Under physiological conditions, the adult brain exclusively depends on glucose oxidation to fuel the high-energy demand for uptake recycling of neurotransmitters and maintenance of ion gradients [1–3]. Local glucose consumption is thus selectively modulated by neuronal activation while being relatively independent of hormonal whole body-derived signals. These principles represent the physiological basis underlying the clinical value of ^18^F-fluoro-2-deoxyglucose (FDG) imaging in different neurodegenerative disorders [4].

Nevertheless, although “brain metabolic independence” has been consistently documented under physiological conditions, a wide literature also reported a measurable response of brain metabolism to severe starvation [5, 6]. This condition can be frequently encountered in patients with Alzheimer’s disease in whom the cognitive impairment can often result in prolonged reduction in food intake with consequent body weight loss.

From the clinical point of view, this impairment contributes to disease progression, particularly in advanced age, as indicated by large epidemiologic studies [7–9]. From the methodological point of view, the possible consequences on diagnostic accuracy of FDG imaging are less certain. In fact, the reduction in glucose availability, combined with the increase in circulating levels of beta-hydroxybutyrate (BHB) and acetoacetate induced by starvation, can switch brain metabolism from a preferential (if not exclusive) glycolytic pattern to a prevalent oxidation of ketone bodies [1, 2, 10, 11]. The consequent reduction in CMRGlu might obviously decrease FDG retention thus asking for dedicated procedures to optimize image quality and counting statistics. On the other hand, the metabolic shift might modify neuronal gene expression profile, promoting the appearance of GLUT carriers and hexokinase isoforms with different affinities for glucose and FDG. This condition might alter the ratio between glucose consumption (CMRGlu) and its index provided by FDG uptake (CMRGlu*) thus modifying the lumped constant value [12, 13]. Finally, the systemic adaptation to food deprivation decreases glucose disposal and FDG sequestration in the whole body, protracting tracer persistence in the bloodstream as to preserve or even increase brain FDG uptake indexed by SUV. The present study aimed to define whether and how the interplay among these three factors induced by starvation interferes with brain FDG uptake and thus with the diagnostic accuracy of PET/CT imaging in neurodegenerative diseases.

## Methods

### Animal models

Six-week-old BALB/c female mice (The Charles River Laboratories, Italy) were housed in sterile enclosures under specific pathogen-free conditions. The 30 mice were divided into two groups: with 15 animals, the “control” group were kept under standard conditions and exposed to fasting for 6 h before the study; the remaining animals were submitted to 48 h of starvation (“STS,” absence of food and free access to water) before imaging. In each group, nine mice were submitted to micro-PET imaging while six mice were sacrificed for the ex vivo studies and thus for measurement of FDG uptake (*n* = 3) or biochemical analyses (*n* = 3), respectively.

### Experimental micro-PET scanning protocol

In vivo imaging was performed according to our validated procedure [14]. Anesthesia was induced by intraperitoneal administration of ketamine (100 mg/kg) and xylazine (10 mg/kg). Capillary glucose level and body weight were measured, and mice were positioned on the bed of a dedicated micro-PET system (Albira, Bruker Inc., USA). A dose of 3–4 MBq of FDG was then injected through a tail vein, soon after the start of a list mode acquisition lasting 50 min.

### Image processing

List data were divided according to the following framing rate: 10 × 15 s, 5 × 30 s, 2 × 150 s, 6 × 300 s, 1 × 600 s, and then reconstructed using a maximal likelihood expectation maximization method (MLEM). Two nuclear doctors unaware of mouse allocation drew a volume of interest (VOI) in the left ventricular chamber to plot the time-concentration curve in arterial blood throughout the whole acquisition (input function). Whole body FDG clearance (in ml × min^−1^) was calculated using the conventional stochastic approach as the ratio between injected dose and integral of input function, fitting the last 20 min with a mono-exponential function [15]. In vivo CMRGlu* was estimated according to Gjedde-Patlak [16] graphical analysis by using the routine of dedicated software (PMOD, Zurich, Switzerland) with lumped constant value set at 1. On these parametric maps, two VOIs were drawn to estimate the average brain (CMRGlu*) and skeletal muscle (SM-MRGlu*) in nMol × min^−1^ × g^−1^. These same VOIs were thus transferred on the last 600-s frame to estimate FDG standardized uptake value (SUV). According to the same procedure, at regional analysis, cortical and cerebellar CMRGlu* and SUV were estimated to calculate the cortical/cerebellum ratio in the two studied subgroups of animals.

### Ex vivo experiments

For “ex vivo” evaluation, each brain was harvested soon after sacrifice, stuck in the outer ring of a Petri dish with octyl-cyanoacrylate (Dermabond, Ethicon, USA), and covered with 2 mL solution collected from an input vial containing 3 mL of DMEM medium (12.5 mM glucose) with a known FDG concentration (1 MBq/mL). Time-activity curve (TAC) of tracer uptake was thus plotted using the Ligand Tracer White device (Ridgeview, Uppsala, Se) [17, 18]. Briefly, this instrument consists of a beta-emission detector and a rotating platform harboring a standard Petri dish. The rotation axis is inclined at 30° from the vertical, so that the medium covers the dish nadir while the detector points at its zenith. All experiments consisted of 45 periodic rotations lasting 1 min and divided into four intervals: (*a*) brain kept for 25 s in the system nadir and thus fully immersed in the incubation medium, (*b*) 5-s 180° counter-clockwise rotation, (*c*) brain kept for 25 s under the detector at the system zenith and, finally, and (*d*) 5-s 180° counter-clockwise rotation for cycle restart. At each cycle, the detector measures background and target counting rates (in counts per second, CPS) in phases *a* and *c*, respectively. FDG brain TAC was thus obtained by subtracting the background counting rate from the corresponding target value [19].

At the end of the experiment, an aliquot of 0.5 mL was sampled both from input vial and from Petri dish (output) to measure glucose concentration (mM) and total FDG activity (MBq). Brain TAC was thus normalized by multiplying brain counting rate at each time *t* (BCR(t)) for the following factor:

1 $$ \mathrm{BFD}(t)=\mathrm{BCR}(t)\times \frac{1}{{\mathrm{BCR}}_{\left(45\ \min \right)}}\times \frac{\left({A}_{\mathrm{input}}-{A}_{\mathrm{output}}\right)\kern0.50em }{A_{\mathrm{input}}} $$

where BFD(*t*) represents the fraction of the dose present in the brain at each time *t*, BCR_(45 min)_ represents the brain counting rate in the last minute, *A*_input_ and *A*_output_ represent FDG activity in MBq at experiment start and end, respectively.

The closed system nature of the Ligand tracer permitted us to consider the input function (IF) as:

2 $$ \mathrm{IF}(t)=1-\mathrm{BFD}(t) $$

BFD(*t*) and IF(*t*) were thus used according to Patlak graphical analysis, assuming the volume invariance of both incubation medium and the brain, during the experiment, respectively. The regression line was defined as:

3 $$ \frac{{\mathrm{BFD}}_t}{{\mathrm{IF}}_t}=a\ \frac{\int_0^t{\mathrm{IF}}_t dt}{{\mathrm{IF}}_t}+b $$

This curve was analyzed in order to verify the expected accumulation kinetics of FDG; the slope *a* was identified by least squares’ definition of regression line and multiplied for input glucose level to estimate CMRGlu*, with the star denoting the FDG-based measurement of CMRGlu, according to the original definition of Sokoloff et al. [20]. By contrast, ex vivo CMRGlu (in nMol × min^−1^) was measured by the equation:

4 $$ \mathrm{CMRGlu}=\left({\mathrm{Glucose}}_{\mathrm{input}}-{\mathrm{Glucose}}_{\mathrm{output}}\right)\kern0.5em \left(\frac{\mathrm{nanoMol}}{\mathrm{mL}}\right)\times \kern0.5em \frac{2}{45}\kern0.5em \left(\frac{\mathrm{ml}}{\min}\right) $$

where glucose represents glucose concentration (nM), 2 is the volume of used DMEM, and 45 is the experiment duration.

### Ex vivo imaging

Soon after the end of the ex vivo experiment, brains were washed and frozen in isopentane chilled with dry ice for sectioning with a cryomicrotome in slices 100 μM thick. At least three sections per brain were placed on a microscope slide and exposed to an imaging plate (Cyclone, PerkinElmer, USA) that provides an image resolution of 100 μm. Exposure time was optimized to 5 min. Thereafter, brain sections were stained with hematoxylin/eosin and photographed by inverted optical microscope. No measurement of radioactivity content was attempted, while autoradiography images were co-registered with the histologic staining using ImageJ software.

### Brain homogenate analysis

For biochemical analyses, brains were homogenized in phosphate-buffer saline (PBS) solution with a Potter-Elvehjem homogenizer. Proteins concentration was performed by Bradford analysis [21]. The samples were sonicated for 10 s in ice.

Western blot experiments were performed accordingly to the standard procedure using 50 μg proteins for each sample. Enzymatic assays were performed spectrophotometrically in a double-beam spectrophotometer (UNICAM UV2, Analytical S.n.c., Italy) using 100 μg of protein for each sample.

Activities of hexokinase (HK), phosphofructokinase (PFK), glucose-6-phosphate dehydrogenase (G6PD), G6Pase, and Complex I (NADH-ubiquinone oxidoreductase) were assayed according to the methods in our previously validated procedure [14]. β-Hydroxy-butyrate-dehydrogenase (BHBDH) activity was evaluated following reduction of NAD^+^ at 340 nm using a solution of 200 mM Tris-HClpH 8, 2 mM NAD^+^, and 30 mM β-hydroxybutyrate. Gluthatione reductase activity was evaluated spectrophotometrically, at 405 nm, using Glutathione Reductase Assay Kit (Abcam: ab83461) following the manufacturer’s instructions. Real-time PCR evaluation was performed according to the standard procedures of our lab [22].

### Value *K*_m_ and *V*_max_ for HK

Hexokinases (HK) Michaelis-Menten kinetics was evaluated for in the presence of glucose and 2-deoxyglucose (2DG; Sigma-Aldrich, Saint Louis, MO, USA). The kinetic characterization of HK was determined at pH 7.4 and 25 °C, by coupling hexose phosphorylation to the reduction of NADP, recording the change in absorbance at 340 nm. The studied initial concentrations were 0.05, 0.1, 1, 5, and 200 mM for glucose and 0.3, 5, 50, 100, and 200 mM for 2DG. To avoid the substrate selectivity, G6PD was substituted with hexose-6P-dehydrogenase (H6PD), as an enzyme able to process both hexoses. *V*_max_ (the maximum rate achieved by the system) and *K*_m_ (the Michaelis-Menten constant indicating the substrate concentration at which the reaction rate is *V*_max_/2) were determined by Lineweaver-Burk double reciprocal plots.

### Statistical analysis

Data are presented as mean ± standard deviation (SD). For comparison between different groups, the null hypothesis was tested by Student’s *t* test for paired or unpaired data, as appropriate. Significance was considered for *p* values *p* < 0.05. Statistical analyses were performed using SPSS software 15.0 (Chicago, IL, USA).

## Results

### Micro-PET analysis of in vivo brain response to starvation

STS caused a significant reduction in body weight (12.7 ± 0.3 vs 16.9 ± 0.9 g, in starved mice and controls, respectively, *p* < 0.001) while its effect on serum glucose level was less pronounced (3.78 ± 1.65 vs 4.78 ± 1.62 mmol/L, respectively, *p* = 0.15). Blood clearance of FDG was significantly lower in starved mice with respect to control ones (0.03 ± 0.01 vs 0.05 ± 0.01 mL × min^−1^, *p* < 0.01).

**Fig. 1:**
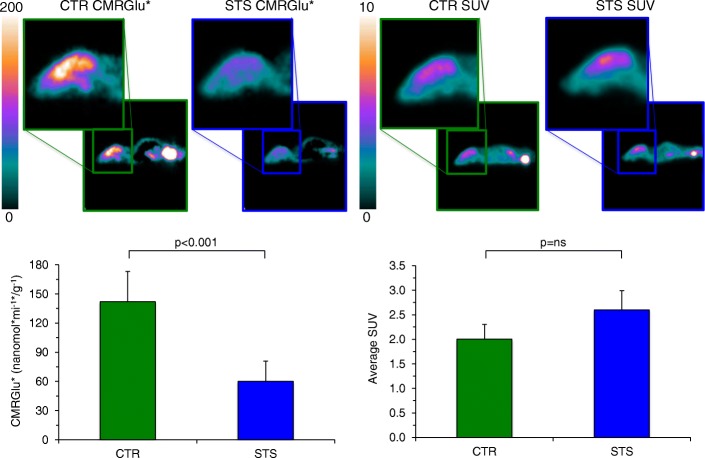
In vivo effect of STS on brain metabolism. In the upper part, on the left, brain parametric maps of representative control (CTR) and 48-h starved (STS) mice are shown; representative images of basal metabolic activity of the whole brain expressed in average standardized uptake value (SUV) are shown on the right. In the bottom, graphs of whole cerebral metabolic rate of glucose (CMRGlu*; nmol × min^−1^ × g^−1^) and SUV are displayed, respectively. Results indicate mean ± SD of nine mice per group; green bars represent control value, and blue bars represent STS value

Compartmental analysis of dynamic micro-PET scans documented a significant response of brain metabolism to STS. In fact, both average slope of Patlak regression line (0.013 ± 0.007 vs 0.027 ± 0.010 min^−1^, respectively, *p* < 0.001) and CMRGlu* (46.1 ± 23.3 vs 119.5 ± 40.2 nmol × min^−1^ × g^−1^, respectively, *p* < 0.001) were lower in starved than in control mice (Fig. 1). By contrast, the STS-related drop in whole body glucose disposal, and the consequent prolongation of tracer availability in the bloodstream, preserved the FDG uptake in the brain, whose average SUV was even (though not significantly) higher in STS than in control mice (2.59 ± 0.36 vs 2.00 ± 0.66, respectively, *p* = 0.14, Fig. 1). At regional analysis, CMRGlu* and SUV cortical/cerebellum ratio remained remarkably stable in both STS and control mice.

**Fig. 2:**
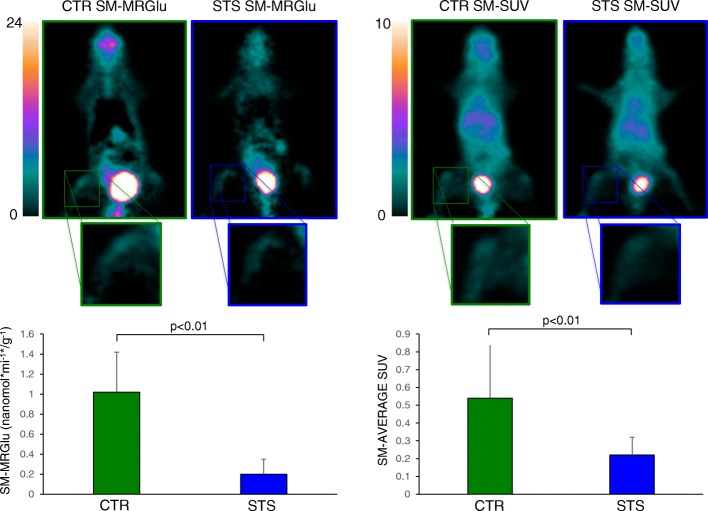
In vivo effect of STS on skeletal muscle metabolism. In the upper part, on the left, skeletal muscle parametric maps of representative control (CTR) and 48-h starved (STS) mice are shown; representative images of basal metabolic activity of hind limbs skeletal muscle expressed in average standardized uptake value (SUV) are shown on the right. In the bottom, graphs of hind limbs skeletal muscle metabolic rate of glucose (SM-MRGlu*; nmol × min^−1^ × g^−1^) and SUV are displayed, respectively. Results indicate mean ± SD of nine mice per group; green bars represent control value, and blue bars represent STS value

This metabolic response was largely different in skeletal muscles (SM) as confirmed by the marked decrease in both hind limbs’ SM-MRGlu* (0.2 ± 0.15 vs 1.02 ± 0.4 nmol × g^−1^ × min^−1^ respectively, *p* < 0.01) and average SUV (0.22 ± 0.1 vs 0.54 ± 0.3, respectively, *p* < 0.01) in starved mice with respect to controls (Fig. 2).

### Ex vivo evaluation of CMRGlu and CMRGlu*

**Fig. 3:**
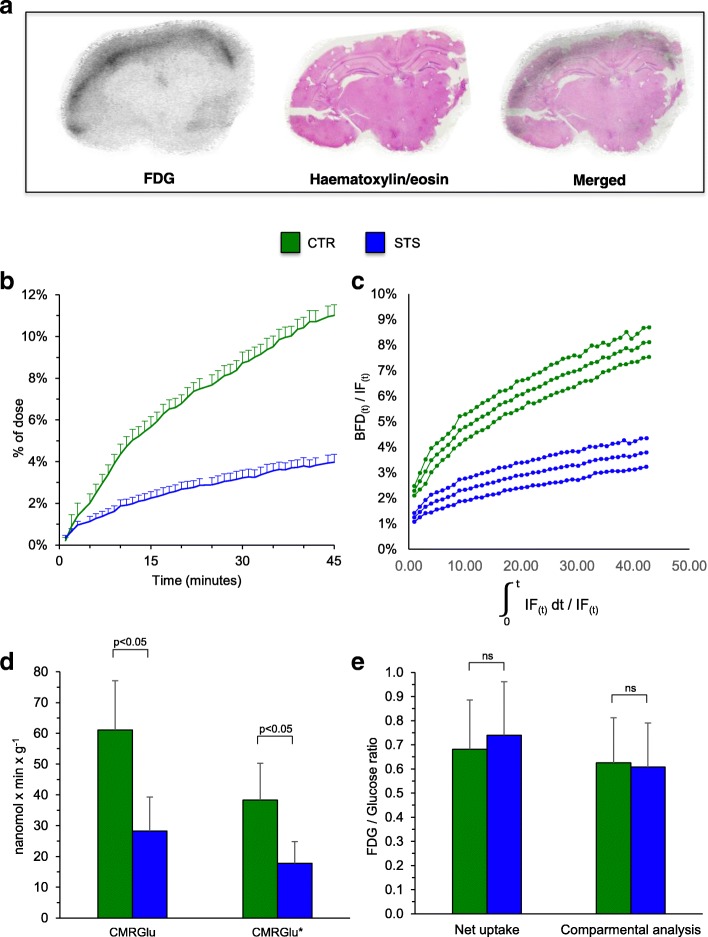
Ex vivo evaluation of CMRGlu and CMRGlu*. **a** The autoradiography (first image), its co-registration with hematoxylin/eosin staining (second picture) and the merging of the two results (third image). **b** The FDG time-activity curves expressed in percentage of dose. **c** The Patlak regression plot. In each graph, control (CTR) mice are represented in green and starved (STS) mice in blue. **d** The CMRGlu (left) and CMRGlu* (right) values expressed in nmol × min^−1^ × g^−1^. **e** The lumped constant value obtained as CMRGlu*/CMRGlu

Autoradiography and its co-registration with hematoxylin/eosin staining documented the expected selectivity of tracer uptake in the gray matter, confirming the viability of all six studied brains during the 45-min incubation (Fig. 3a).

The ex vivo evaluation confirmed the STS effect on brain metabolism documented in vivo. Despite the identical extracellular environments, previous 48-h food deprivation markedly reduced all indexes of brain hexose intake. In fact, fractional drop of glucose concentration in the incubation medium was markedly lower in starved than in control mice (0.05 ± 0.02 vs 0.11 ± 0.04%, respectively, *p* < 0.05, Fig. 3b). A similar observation also applied to the fractional loss of FDG (Fig. 3c) from the incubation medium that was almost halved in brains explanted from starved mice (0.037 ± 0.015%) with respect to control ones (0.075 ± 0.02%; *p* < 0.01).

Obviously, this same response was documented by the estimation of metabolic rates. In fact, CMRGlu was 61.1 ± 16.1 nmol × min^−1^ in control preparations and decreased to 28.27 ± 12.7 nmol × min^−1^ in STS ones (*p* < 0.01). This response closely agreed with the corresponding CMRGlu*. In all brains, FDG time-activity curves showed a linear increase during the whole experiment (Fig. 3d) and thus confirmed the expected accumulation kinetics of FDG. Similarly, Patlak regression plot showed a good correlation with *r* values always ≥ 0.97. Again, previous STS decreased CRMGlu* from 38.3 ± 11 to 17.8 ± 7.2 nmol × min^−1^ (*p* < 0.05, Fig. 3d).

As a consequence, lumped constant value was not affected by nutritional status. In fact, the ratio between fractional removals of FDG and glucose from the medium was superimposable in control and STS brains as well as the ratio CMRGlu*/CMRGlu (Fig. 3e).

### Starvation effect on determinants of glucose and FDG entrapment

**Fig. 4:**
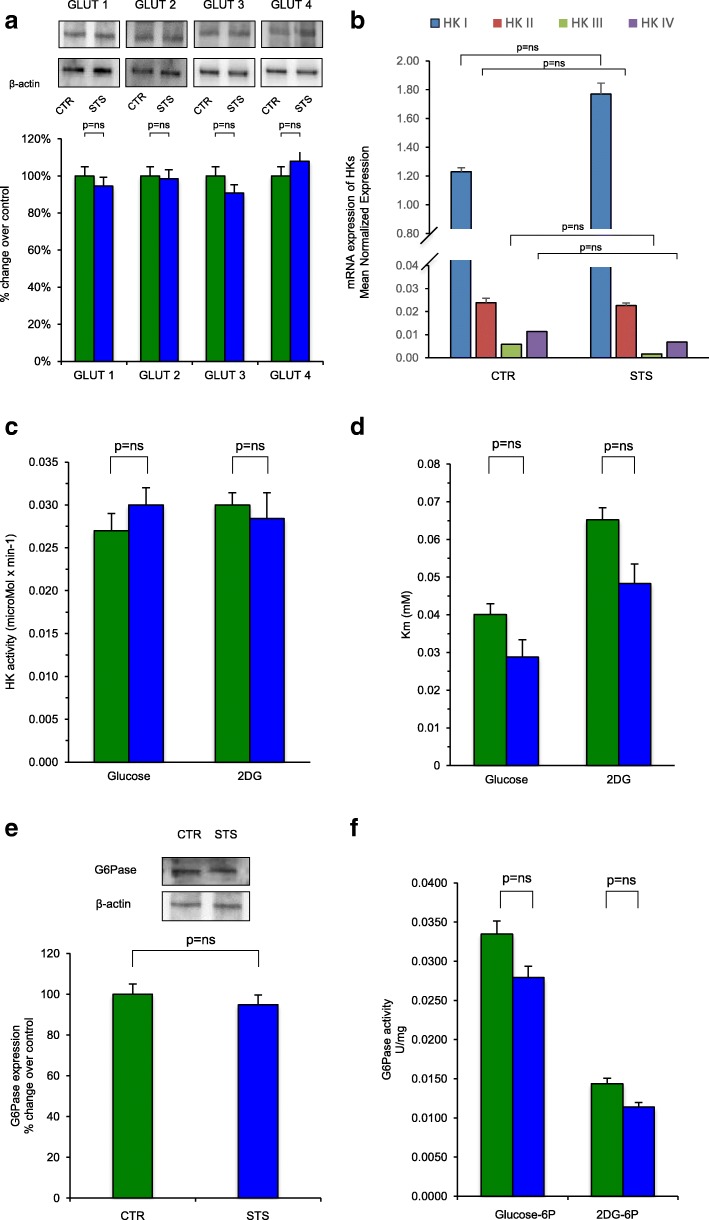
STS effect on determinants of glucose and FDG entrapment. **a** The western blot analysis against GLUT1, GLUT2, GLUT3, and GLUT4 performed in brain homogenate of control (CTR, green bars) and of starved mice (STS, blue bars). Protein levels of the different GLUT isoforms were remarkably stable regardless of nutritional status. Data were expressed in percentage change over control ± SD of at least three experiments. The values have been normalized on β-actin. **b** The HK gene expression profiling of brain homogenates in CTR (left) and STS mice (right) was obtained by real-time PCR. The four different isoforms have been analyzed: HK I (blue bar), HK II (red bar), HK III (green bar), and HK IV (purple bar). Results are expressed in mean normalized expression ± SD. Nutritional status did not affect this expression profile. **c** The HK activity of brain homogenate in CTR mice (green bar) and in STS mice (blue bar) in the presence of glucose (left) and 2DG (right). Data are expressed in μmol × min^−1^ ± SD of at least three experiments. **d** HK *K*_m_ expressed determined by Lineweaver-Burk double reciprocal plots. Data are expressed in mM ± SD of at least three experiments. **e**, **f** the western blot analysis against G6Pase and the G6Pase activities, respectively. In both graphs, CTR group is represented in blue and the STS mice in green. Western blot results are expressed in percentage change over control ± SD of at least three experiments. Values have been normalized on β-actin. The G6Pase activity was assayed in the presence of glucose-6P (left) and 2DG-6P (right), and the values are expressed in U × mg of proteins^−1^ ± SD of at least three experiments

The marked reduction in glucose consumption and FDG uptake induced by STS was not explained by changes in the membrane hexose carriers. In fact, protein levels (Fig. 4a) of the different GLUTs (GLUT 1–2–3–4) were remarkably stable regardless of nutritional status.

A similar response was documented for HK isoforms. Coherently with the literature [23], HK gene expression profiling of brain homogenates documented high levels of mRNA encoding for HK isoform I, with only trace signals for isoforms HKII, HKIII, and HKIV. Nutritional status did not significantly affect neither HK expression profile (Fig. 4b) nor kinetic features of hexose phosphorylation. In fact, estimated *V*_max_ (Fig. 4c) and *K*_m_ (Fig. 4d) of HK-related catalysis were only trivially affected by STS for both glucose and 2DG confirming the theoretical basis for the observed lumped constant invariance.

Although the expression of G6Pase was very low in controls, Western blot analysis demonstrated a slight and not significant reduction of protein amount in starved brains (Fig. 4e). On the other hand, the activity of G6Pase were found to be extremely low in brain homogenates under normal conditions and remained unchanged regardless nutritional status (Fig. 4f).

**Fig. 5:**
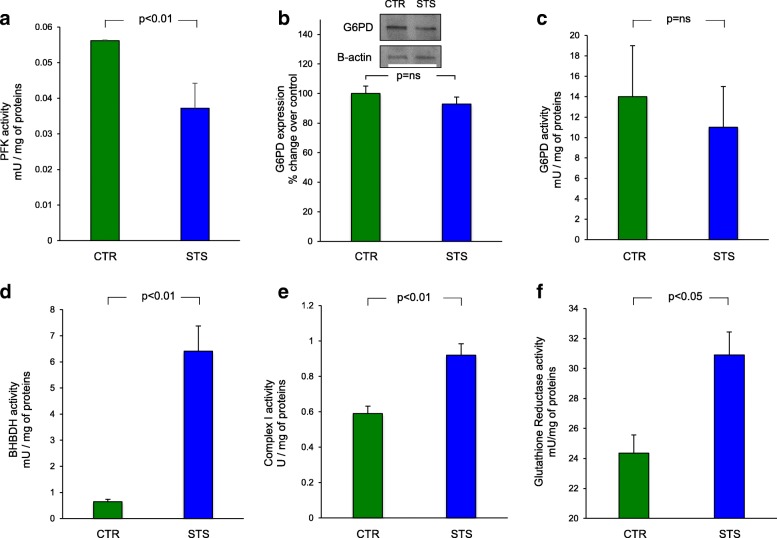
Metabolic machinery of starved brain. In each panel, green bars represent the brain homogenate of control (CTR) mice and the blue bars represent the brain homogenate of starved (STS) mice. **a** The PFK activity expressed in mU × mg of proteins^−1^ ± SD of at least three experiments. The catalytic activity was reduced in STS brain homogenate with respect to CTR

On the contrary, STS significantly reduced the catalytic activity of PFK, the rate-limiting enzyme of glycolysis (3.7 ± 0.6 × 10^−2^ vs 5.6 ± 0.1 × 10^−2^ mU/mg of proteins in STS and control brains respectively, *p* < 0.01, Fig. 5a). In agreement with this response, lactate release slightly decreased (though not significantly) from 28 ± 9 in controls to 19 ± 6 nmol × min^−1^ in STS mice (*p* = 0.20). The evident reduction in glycolytic flux was not counterbalanced by any response of PPP since its regulator G6PD showed constant mRNA levels (0.35 ± 0.01 vs 0.30 ± 0.02 mean normalized expression in control and STS brains, respectively, *p* = ns), protein abundance (Fig. 5b), and activity (Fig. 5c).

As expected, the reduced hexose avidity induced by STS was paralleled by a metabolic shift from a prevalent glycolytic pattern to a preferential oxidation of ketone bodies [24]. This concept was confirmed by the evaluation of BHBDH activity that markedly increased in STS brain lysates with respect to control ones (6.41 ± 0.96 vs 0.64 ± 0.09 mU/mg of proteins, respectively, *p* < 0.01, Fig. 5d). Similarly, it was corroborated by the response of respiratory Complex I, whose activity significantly increased in STS brain with respect to controls (0.92 ± 0.06 vs 0.59 ± 0.04 U/mg of proteins, respectively, *p* < 0.01; Fig. 5e). Finally, and in line with the respiratory burst intrinsically associated with ketone bodies catabolism, glutathione reductase showed a significant increase after STS (Fig. 5f).

## Discussion

The present study documents that starvation markedly decreases brain glucose consumption without altering measured FDG SUV in mouse experimental models. This apparent paradox does not reflect any change in lumped constant since the ratio between tracer uptake and glucose intake remains remarkably constant regardless of nutritional condition. Rather, it is explained by the metabolic response of the whole body: the decrease in FDG sequestration by the skeletal muscle is as profound as to prolong tracer persistence in the bloodstream and thus its availability for brain uptake.

### Biochemical considerations

In the present study, control CMRGlu* was comparable to the data reported by Kreissl et al. in mice exposed to a similar fasting duration [25]. However, this value was almost halved after 48-h STS. In agreement with the evident decrease in PFK [26] activity, this response confirms previous studies showing a profound deceleration in brain glycolytic rate after prolonged food deprivation and weight loss both in humans [5, 6] and rodents [27]. A similar consideration applies to the almost fivefold increase of BHBDH activity in starved brains. This observation agrees with the acknowledged brain metabolic response to STS in which the preferential utilization of ketone bodies [28] accelerates cell respiration as confirmed by the enhancement in mitochondrial Complex I function. Similarly, the relatively low OXPHOS efficiency [24] of ketolysis, and the consequent rise in reactive oxygen species, nicely explains the increase in glutathione reductase activity [29] as a basic cell response to oxidative stress.

Accordingly, the observed halving of CMRGlu* induced by STS is coherent with a marked reduction in brain glycolytic flux compensated by an accelerated ketolysis. Although blood levels of ketone bodies were not tested in our models, this concept is largely confirmed by the ex vivo part of our study. Explanted STS brains were immersed in a medium with high glucose concentrations and without any other competing substrate. Under this condition, the reduced avidity for glucose and FDG persisted in all brains harvested from STS mice suggesting a profound shift in the metabolic machinery of studied nervous tissues.

### Methodological considerations.

Besides its biochemical relevance, the pilot ex vivo evaluation provides a direct experimental and theoretical evidence of lumped constant stability under severe food deprivation. Previous attempts to approach this issue in vivo obtained conflicting results: the compartmental analysis applied by Redies and coworkers [6] reported an STS-related reduction in lumped constant that was not confirmed by Hasselbalch et al. [30] who compared CMRGlu (measured by Fick method) with CMRGlu* (measured by brain kinetics of FDG uptake).

Our ex vivo approach permitted us to couple tracer-based estimation of CMRGlu* with the direct measurement of CMRGlu. The ratio between these two variables remained remarkably stable in brains harvested from normally fed or starved mice. The stability of lumped constant was confirmed by the invariance of its theoretical determinants. According to the original Sokoloff statement, the ratio between glucose intake and 2DG uptake reflects the different affinities for the two hexoses of factors regulating transmembrane transport, hexose phosphorylation and de-phosphorylation: GLUT expression (lambda) was not affected by STS and, similarly, G6Pase activity (phi) on both glucose and 2DG was unchanged regardless nutritional condition. Moreover, direct measurements of *K*_m_ and *V*_max_ for both glucose and 2DG documented an absent effect of STS on HK isoforms affinity for the two hexoses.

**Fig. 6:**
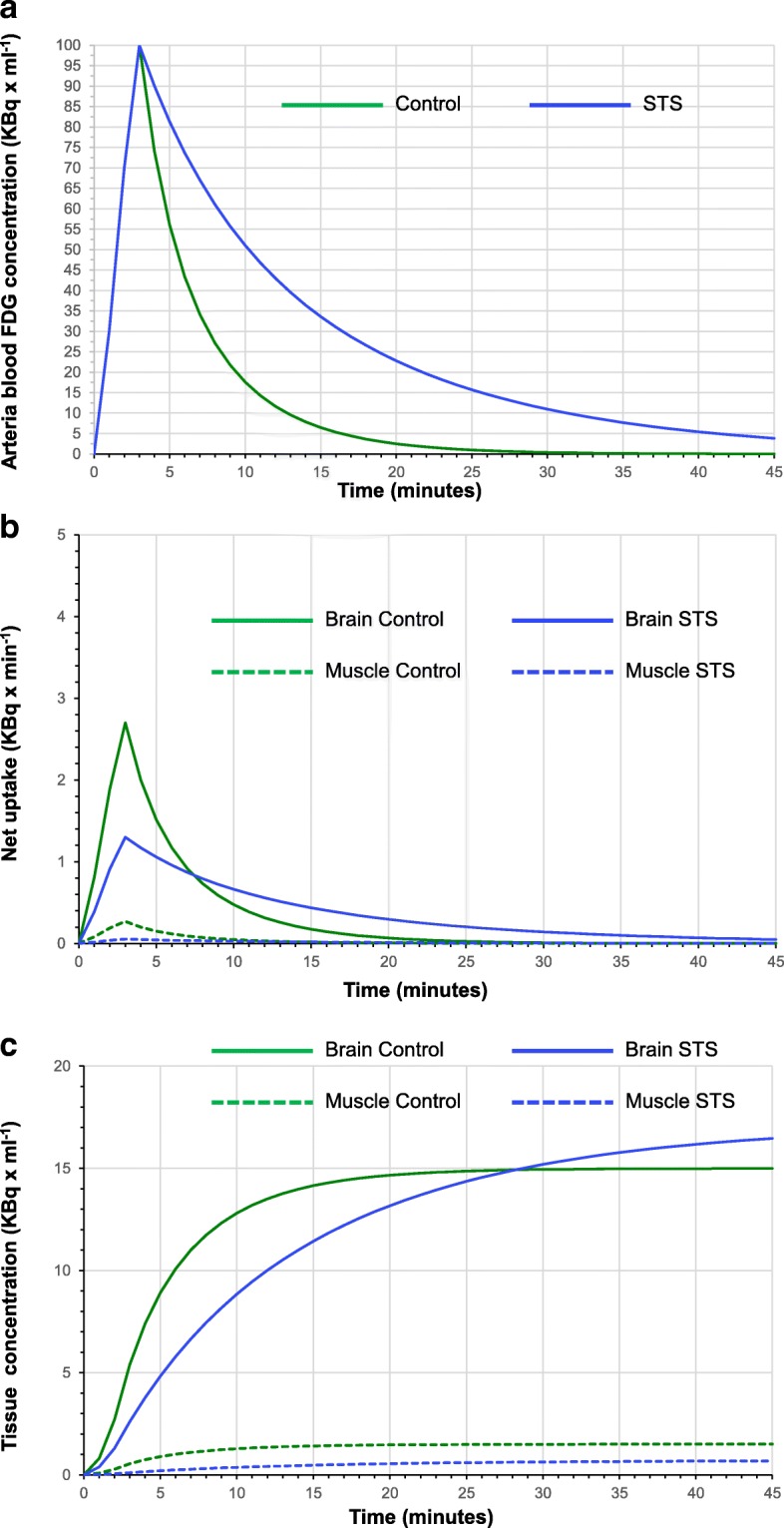
Theoretical model of FDG uptake in the brain and skeletal muscle. This cartoon represents a theoretical model for FDG uptake in the brain and skeletal muscles in control (green) and STS (blue) mice. **a** FDG arterial concentration as predicted by the corresponding average clearance value measured in the two groups: the decreased tracer sequestration by the whole body prolongs tracer availability in the bloodstream. **b** The instantaneous uptake (KBq × min^−1^). The curves are defined assuming an identical flow rate in the two conditions, with final uptake values only justified by the interaction between extraction fraction and tracer availability. The brain and skeletal muscle are represented by solid and dotted lines, respectively. **c** Time-concentration curves in the two tissues and points out the progressive increase in STS brain as a consequence of the prolonged tracer availability. Despite a virtual halving of extraction fraction, the prolonged uptake phase eventually results in preserved FDG uptake a later time. This phenomenon is markedly less evident in the skeletal muscle due to the relatively more severe (fivefold vs twofold) reduction in glucose consumption

Accordingly, “ex vivo” experiments indicate that the observed response of CMRGlu* to STS did reflect a profound reduction in brain glycolytic rate. The preserved (or even slightly increased) brain FDG SUV observed in starved mice was instead explained by the relatively more profound impairment in glucose utilization by the whole body of starved mice. This response has been already reported in the literature as a possible consequence of the increased blood concentration of ketone bodies under starvation [31]. In agreement with this study, the severe (almost fivefold) reduction in skeletal muscle avidity for FDG profoundly altered tracer kinetics virtually halving its blood clearance (Fig. 6a). The decreased CMRGlu halved the tracer extraction fraction in STS brain (Fig. 6b). As a consequence, FDG brain retention increased more slowly in starved mice with respect to control ones. However, the longer duration of tracer availability prolongs the uptake process up to values higher with respect to control SUVs (Fig. 6c).

Altogether, the present study indicates that STS actually impairs brain glycolytic flux as documented by the similar decrease in both CMRGlu and CMRGlu*. This response reflects an adaptation in the enzymatic machinery of cells populating the central nervous system and persists under exposure to high glucose concentration without the competition of any other metabolite.

## Conclusions

From the methodological point of view, the present findings confirm the theoretical limitations of brain SUV as a surrogate of CMRGlu* in estimating brain glucose consumption. However, the clinical interpretation of FDG imaging mostly relies on the identification of relative metabolic inhomogeneities as markers of neurodegenerative disorders. The virtually absent response of FDG SUV to STS thus further confirm the adequacy of current procedures even in underfed patients provided that time gap between injection and imaging is carefully respected. Defining the regional distribution of STS effect throughout the different cortical areas was not possible with our experimental model. The relevance of this limitation is indicated by documented heterogeneity in metabolic response to food deprivation in the different brain structures [32, 33]. Nevertheless, the severe brain metabolic impairment induced by STS indicates a possible predictive power of regional CMRGlu*. This potential might configure dynamic PET studies to evaluate the link between nutritional status and neurodegeneration.
